# Definitive and adjuvant radiotherapy for sinonasal squamous cell carcinomas: a single institutional experience

**DOI:** 10.1186/s13014-015-0496-3

**Published:** 2015-09-17

**Authors:** Sumerya Duru Birgi, Mark Teo, Karen E. Dyker, Mehmet Sen, Robin J D Prestwich

**Affiliations:** Department Of Clinical Oncology, St. James’s Institute of Oncology, Level 4, Bexley Wing, Beckett Street, Leeds, West Yorkshire LS9 7TF UK

## Abstract

**Background:**

The aim of this study was to evaluate the disease outcomes of patients treated with definitive and adjuvant radiotherapy for squamous cell carcinomas of the nasal cavity and paranasal sinuses in a single institution.

**Methods:**

Between 2007–2012 patients were retrospectively identified from electronic databases who had undergone surgery and adjuvant radiotherapy or definitive radiotherapy for sinonasal squamous cell carcinomas with curative intent.

**Results:**

Fourty three patients with sinonasal squamous cell carcinoma were identified (22 nasal cavity, 21 paranasal sinuses). 31/43 (72 %) had T3 or T4 disease; nodal stage was N0 in 38, N1 in 4, Na/b in 0 and N2c in 1 patient. Median age was 67 years (range 41–86). 18 (42 %) received definitive and 25 (58 %) adjuvant radiotherapy. Radiotherapy was delivered using either conventional radiotherapy (*n* = 39) or intensity modulated radiotherapy (*n* = 4). Elective neck radiotherapy was delivered to two patients. Chemotherapy was delivered to 6/43 (14 %) of patients. Two-year local control, regional control, distant metastases free survival, progression free survival, cause specific survival and overall survival were 81 %, 90 %, 95 %, 71 %, 84 % and 80 % respectively. There was no significant difference in outcome comparing patients who underwent surgery and adjuvant radiotherapy with patients receiving definitive radiotherapy (2 year locoregional disease free survival 75 % and 70 % respectively, *p* = 0.98). Pooly differentiated tumours were significantly associated with inferior disease outcomes. Local, regional, combined local and regional, and distant failure occurred in 7 (16 %), 3 (7 %), 1 (2 %) and 2 (5 %) of patients; all 3 regional recurrences were in patients with nasal cavity squamous cell carcinomas who had not undergone elective neck treatment.

**Conclusions:**

Definitive or adjuvant radiotherapy provides an effective treatment for sinonasal malignancies. The main pattern of failure remains local, suggesting the need for investigation of intensified local therapy. Whilst remaining uncommon, the cases of regional failure mean that the merits of elective lymph node treatment should be considered on an individual basis.

## Introduction

Sinonasal malignancies are rare [1] and include a wide range of histopathological cell types [2, 3]. The anatomical structure of the sinuses allows asymptomatic growth until local structures/organs are invaded; most patients therefore present with locally advanced disease [4]. Determining optimal management continues to be challenging due to the rarity of the disease and proximity to multiple critical structures including the optic nerves, chiasm, eyes and brainstem. Management choices have largely been informed by single institution retrospective series. Treatment approaches have generally involved surgery followed by adjuvant radiotherapy, or definitive radiotherapy for patients for whom complete surgical excision is unlikely, co-morbidity or patient preference [4, 5].

Controversies remain in the management of sinonasal squamous cell carcinomas. Local failure is the major pattern of relapse and has guided treatment approaches [6]. However, there is variation in practice regarding the necessity for elective treatment of a clinically/radiologically N0 neck [6, 7]. In addition, by contrast with more common cancers sites within the head and neck region, there is not a strong evidence based for the role of chemotherapy; this at least partly relates to the rarity of the disease. The use of chemotherapy has been generally at clinician discretion in several series [2, 5, 8]

In this study, we report the disease outcomes and patterns of failure for patients with sinonasal squamous cell carcinomas treated with radiotherapy in the post-operative or definitive setting.

## Materials and methods

### Study design

A retrospective study was performed using patient records, radiotherapy treatment plans and diagnostic imaging on 43 patients who had been treated with either definitive or adjuvant radiotherapy between 2007–2012 for sinonasal squamous cell cancers. Patients were excluded if radiotherapy was delivered with palliative intent. Patients were staged according to the American Joint Committee on Cancer 7th Edition [9]. Clinical and radiological staging was used for patients treated non-surgically, whilst pathological staging was used for patients who had undergone surgery.

### Treatment

#### Surgery

All patients underwent evaluation in a Head and Neck multidisciplinary team to evaluate the feasibility/appropriateness of surgical resection. The type of surgery depended upon primary site, extent of disease, cosmetic considerations and discretion of the surgical team; surgery was always aimed at obtaining a gross total resection.

### Radiotherapy

Three patients with a nasal vestibule cancer were treated with a direct electron field. 39/43 (91 %) of patients were treated with a 3D-conformal photon technique and toward the end of the study period six patients (12 %) were treated with intensity modulated radiothearapy (IMRT). Patients were immobilised with a Perspex mask with a neutral neck position. A CT scan with 2-5 mm slices was acquired. MRI co-registration was not available during the study period. A mouthbite was routinely used to minimise the radiation dose to the lower part of the oral cavity. A compartmental approach to target volume delineation was adopted. For patients receiving definitive radiotherapy a gross tumour volume (GTV) was outlined as primary tumour and clinically and/or radiologically involved lymph nodes. A primary tumour clinical target volume (CTV) was created to include at least GTV + 10 mm and modified to include the whole involved anatomical compartment or sinus ie. all invaded or partly invaded sinuses, modified to anatomical boundaries to exclude air and/or bone without evidence of invasion. For patients who had undergone macroscopically complete surgical resection of the tumour, the same compartmental approach was adopted to delineate the CTV encompassing the resection cavity. Elective treatment of the neck was not routinely performed for clinically node negative disease; neck treatment was delivered for N+ neck disease. Planning target volumes (PTV) were generated by an auto-expansion of 4 mm to CTV structures.

Organs at risk routinely delineated on the planning CT scan included: optic chiasm, brainstem, optic nerves, spinal cord.

Radiotherapy was delivered five fractions per week. The prescription dose was at the discretion of the treating clinician; standard adjuvant doses were 60-66Gy in 30–33 fractions and definitive doses included a hypofractionated regimen of 55Gy in 20 fractions in the early part of the study period, and conventionally fractionated 66-70Gy in 33–35 fractions in the latter part of the study. Standard dose constraints during this study period were: spinal cord <48Gy, optic nerves, brainstem and chiasm <54y, lens as low as achievable.

The 3D-conformal technique was based on either a three-field technique with a heavily weighted anterior portal with two lateral wedged portals or an anterior wedged pair technique depending upon the target volume. IMRT was delivered with a 5–7 angle step and shoot IMRT technique.

All photon treatment was delivered with 6MV photons. Treatment was planned to provide adequate coverage of the target volumes according to ICRU-62 guidelines [10].

### Chemotherapy

Chemotherapy was employed upon clinician discretion, based upon tumour histology and high risk features. Concurrent chemotherapy was with cisplatin 100 mg/m^2^ days 1 and 29. Induction chemotherapy regimens included cisplatin 80 mg/m^2^ day 1 and 5-fluorouracil (5FU) 800 mg/m^2^ days 2–5, three weekly.

### Follow up schedule

Patients were seen at least once per week during radiotherapy treatment. For patients receiving definitive radiotherapy, response assessment imaging with CT and/or MRI was routinely performed 3 months post-treatment. Patients were followed up with physical examination every 6–8 weeks in the first year after treatment, every 3 months for an additional 2 years, and every 6 monthly until discharge at 5 years. Suspected recurrence was detected by clinical examination and radiological examination and biopsy if appropriate.

### Statistical analysis

Statistical analysis was performed using SPSS version 16 (IBM, USA), STATA version 10 (Statacorp, USA), and Prism version 6 (Graphpad, USA). Survival and recurrence outcomes were calculated from the date of their curative treatment (i.e. date of curative surgery, date of first fraction of radiotherapy). The following endpoints were used for assessment: local control rate, regional control rate, locoregional disease free survival (LRDFS), distant metastases free survival (DMFS), progression free survival (PFS), cause specific survival (CSS) and overall survival (OS). For comparison of surgery and adjuvant radiotherapy versus definitive radiotherapy, univariate log-rank survival analysis was performed for each outcome and hazard ratios (HR) and 95 % confidence intervals calculated.

## Results

Fourty three patients with sinonasal squamous cell carcinomas were identified who had received definitive or adjuvant radiotherapy. Patient and disease characteristics are summarised in Table 1. Median follow up was 32 (range 4–102) months. Median age was 67 years (range 41–86). 35/52 (67 %) had T4a or T4b disease.

**Table 1:** Patient and tumour characteristics

Characteristics	Number (*n* = 43)	Percent
Gender		
Male	25	60
Female	18	40
Tobacco use at diagnosis		
Ex smoker	8	19
Current smoker	15	35
No smoker	7	16
Not documented	13	30
Tumour localisation		
Maxillary sinus	20	47
Ethmoid sinus	1	2
Nasal cavity	22	51
Nasal vestibule	10	23
Nasal septum	3	7
Floor and lateral wall	9	21
T stage		
T0	0	0
T1	6	14
T2	6	14
T3	2	5
T4a	23	53
T4b	6	14
N stage		
N0	38	88
N1	4	9
N2a	0	0
N2b	0	0
N2c	1	3
Stage group (AJCC 2010)		
I	6	14
II	6	14
III	2	5
IVa	23	53
IVb	6	14
Histopathology		
SCC	43	100
Histopathological differentiation		
Well differentiated	7	16
Moderately differentiated	18	40
Poorly differentiated	10	23
Undifferentiated	3	7
Not documented	6	14
Nodal involvement		
Yes	3	7
No	40	93
Nodal ECE		
Yes	1	20
No	4	80
Tumor invasion		
Cranial	2	5
Orbital	2	5
Skin	7	16

Treatment details are summarised in Table 2. 25/43 (58 %) of patients had undergone surgery, with a positive resection margin (<1 mm) reported in 13/25 (52 %) and close (<5 mm) margins in 5/25 (20 %). 36/43 (84 %) of patients received radiotherapy via a 3D-conformal technique. Neck radiotherapy was delivered to 3 patients; in one case neck radiotherapy followed neck dissections for N+ disease and in two cases elective neck radiotherapy was delivered to the ipsilateral neck along with treatment of the primary site following surgery for T4N0 squamous cell carcinomas of the maxillary sinus. Chemotherapy was delivered to a total of 6/43 (14 %) of patients. Comparing patients who underwent surgery and adjuvant radiotherapy and patients who received definitive radiotherapy, there were significantly more patients undergoing surgery with T4a disease (*p* < 0.001) and a non-significant trend for more patients with maxillary tumours to undergo surgery (*p* = 0.06). 12/18(67 %) patients who received definitive radiotherapy were documented as having achieved a complete clinical and radiological response to treatment.

**Table 2:** Treatment details

	Number (n) (*n* = 43 unless stated otherwise)	Percentage (%)
Surgery	*n* = 25	58
Open	23	92
Endoscopy	2	8
Unilateral neck dissection	12	48
Bilateral neck dissection	2	8
Margin status	*n* = 25	
Positive	13	52
Close (<5 mm)	5	20
Negative (≥5 mm)	6	24
Unknown	1	4
Perineural invasion	*n* = 25	
Yes	5	20
No	10	40
Not reported	10	40
Lymphovascular invasion	*n* = 25	
Yes	1	4
No	13	52
Not reported	11	44
Radiotherapy		
Electron	3	7
3DCRT	36	84
IMRT	4	9
Radiotherapy		
Adjuvant	25	58
Radical	18	42
Radiotherapy dose		
Radical		
70 Gy/35 fx	5	12
68 Gy/34 fx	1	2
66 Gy/33 fx	2	5
55 Gy/20 fx	10	23
Adjuvant		
66 Gy/33 fx	8	19
64 Gy/32 fx	3	7
62 Gy/31 fx	1	2
60Gy/ 30 fx	7	16
55 Gy/20 fx	6	14
Radiotherapy region		
Primary	40	93
Primary and ipsilateral neck	3	7
Primary and bilateral neck	0	0
Chemotherapy		
Induction	3	7
Concurrent	3	7

**Fig. 1:**
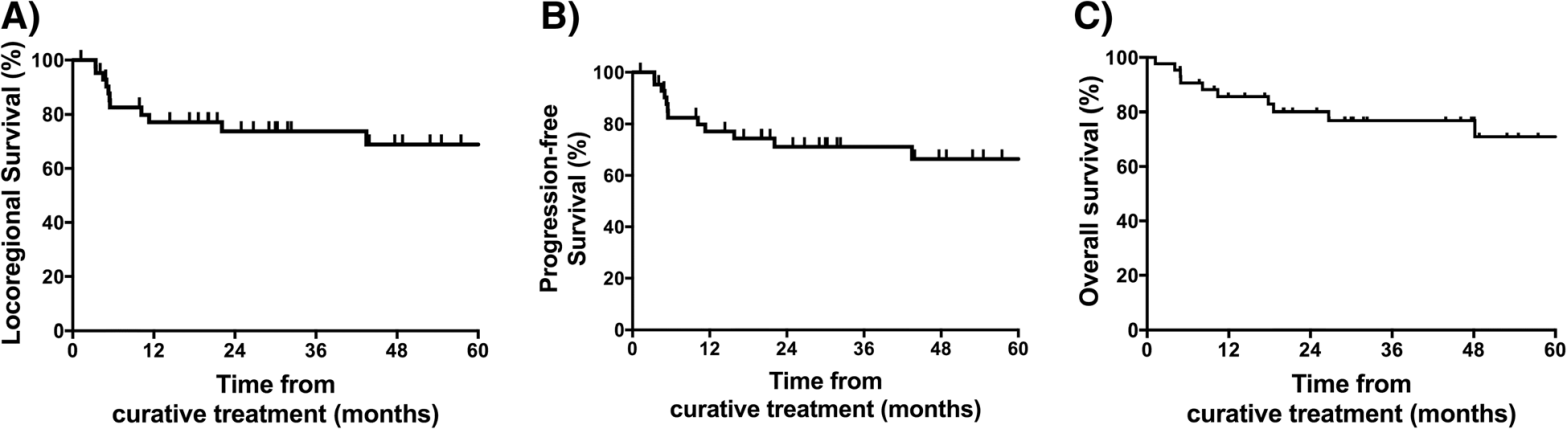
Kaplan Meier curves showing **a** locoregional control, **b** progression free survival and **c** overall survival

The 2-year local control rate, regional control rate, DMFS, PFS, CSS and OS were 81 %, 90 %, 95 %, 71 %, 84 %, and 80 % respectively. 5-year local control rate, regional control rate, DMFS, PFS, CSS and OS were 76 %, 90 %, 95 %, 66 %, 74 %, and 71 % respectively. Locoregional control, PFS and OS are illustrated in Fig. 1. Local, regional, combined local and regional, and distant failure occurred in 7 (16 %), 3 (7 %), 1 (2 %) and 2 (5 %) of patients. Median time to local recurrence was not reached. There was no significant difference comparing outcomes of patients who underwent radical radiotherapy (*n* = 18) and surgery and adjuvant radiotherapy (*n* = 25): 2-year LRDFS was 70 % versus 75 % (non-significant, *p* = 0.98), and 2-year CSS was 94 % versus 79 % (non-significant, *p* = 0.6)). Recurrence patterns are summarised in Table 3. Poorly differentiated tumours were significantly associated with increased risk of developing distant metastases (HR 8.2 (95 % CI1.01-66) *P* = 0.05 and inferior CSS (HR2.31(95 % CI1.06-5.0, *P* = 0.04). A total of eight patients experienced local disease recurrence; one of these cases was in combination with regional failure. Primary tumour site involved nasal cavity in 4, ethmoid sinus in 1 and maxillary sinus in 3 patients respectively; disease stages were T4N0 in 6 patients, T1N0 in one patient and T2N0 in one patient. Four of these patients had initially undergone surgery and adjuvant radiotherapy and four patients were initially treated with definitive radiotherapy (one with concurrent chemotherapy). The two patients with early stage disease at outset underwent successful surgical salvage of local recurrence; the remaining six patients were managed with palliative approaches.

**Table 3:** Patterns of disease recurrence

Recurrence site	Number
Local	
Radical	4
Adjuvant	3
Regional	
Radical	0
Adjuvant	3
Local and regional	
Radical	0
Adjuvant	1
Distant metastasis	
Radical	1
Adjuvant	1

The four patients who experienced a regional lymph node recurrence had all undergone surgery and adjuvant radiotherapy without elective neck surgery or irradiation for squamous cell carcinoma of the nasal cavity; one was a pT2 and the remaining cases pT4 disease. In these four cases one recurrence occurred with simultaneous local recurrence. The pattern of regional recurrences were in level II lymph nodes in one case, level Ib lymph nodes in two cases and facial lymph nodes in one case. Two patients with regional lymph node recurrence underwent salvage surgery and radiotherapy; one of these patients subsequently developed widespread distant metastases whilst the other remains disease free. One patient was not deemed fit for further treatment, and one patient with synchronous local and regional progression received palliative chemotherapy. There were no cases of regional recurrences in patients treated for paranasal sinus cancers.

## Discussion

Sinonasal squamous cell carcinomas present a major therapeutic challenge. In the absence of randomised trials, treatment options for sinonasal malignancies are based on retrospective series by institutions; due to the rarity of the disease, data remains limited. Historical outcomes following 2-dimensional radiotherapy are limited in terms of disease outcomes and treatment-related late morbidity [11–13]. The introduction of 3D-conformal radiotherapy facilitated an improvement in disease and toxicity outcomes [14–16]. More recently the advent of IMRT techniques has allowed improved target volume coverage with a minimisation of OAR doses [17]. Reported outcomes in previous studies vary widely; for example, local control rates and overall survival rates are reported between 21–84 % and 9–89 % at 2 to 5 years follow up [2]. Overall outcomes appear to be superior in more recent series. For example, IMRT series have reported 2-year local control rates of 62–76 % [2–5]; 2-year overall survival in these modern series ranged from 66–89 %. Comparison between series is difficult, due the heterogeneity of tumour site, stage, treatment approaches and duration of follow up. In common with other series, our report includes a both a mixture of primary tumour sites including both nasal cavity and paranasal sinuses [2–5, 8, 18]. Broadly, disease outcomes from our series appear favourable compared with many series and similar to those reported recently by other major institutions [2–4]. The proportion of patients in our series with nasal cavity tumours was high (51 %) compared with other reports (6–28 %, [2–5, 8, 18]). Although there was no significant difference in outcomes between nasal cavity and non-nasal cavity primary sites, this may have influenced outcomes favourably in our predominantly non-IMRT experience due to the comparative ease by which nasal cavity tumours can be treated compared with other paranasal sinus primaries with 3D-conformal radiotherapy.

The conformality provided by IMRT appears to provide increased levels of local control with very low rates of late toxicity [3–5, 8]. Recent series reporting outcomes following IMRT for paranasal sinus malignancies have reported no cases of optic neuropathy with limited follow up [8, 19]. IMRT is now regarded as a standard of care for paranasal sinus cancers [19]. A limitation of our series is that the majority of patients were treated with a 3D-conformal radiotherapy technique; IMRT is now the standard of care within our institution for paranasal sinus tumours.

Patients with a gross tumour resection prior to radiotherapy are associated with favourable outcomes [18, 20, 21]. En bloc removal of macroscopic disease is regarded as the aim of initial treatment. This has been facilitated by ongoing improvements in surgical techniques. There was no significant difference in outcome in patients undergoing definitive radiotherapy and surgery and adjuvant radiotherapy in our series. Although hypothesis generating, these data may be influenced by an imbalance between the groups with an excess of nasal cavity cancers treated non-surgically.

The majority of failures in our series were local recurrences; this is consistent with multiple previous series [2, 3, 8]. This suggests that the focus of improving sinonasal tumour outcomes will continue to be improving local treatment outcomes. The excellent results of recent IMRT series suggest that the improved dose conformality leading to less compromise of target coverage may translate into improved local control. Another potential route to improving the efficacy of treatment is the incorporation of chemotherapy. The role of chemotherapy in the treatment of sinonasal malignancies is uncertain. Only 14 % of patients in our series received either induction or concurrent chemotherapy at the discretion of the treating clinician. Similarly in some [2, 22] but not all series [3–5], a limited proportion of patients were treated with chemotherapy. An extrapolation from other head and neck cancer sites [23] would suggest that the use of concurrent chemotherapy may be advantageous for squamous cell cancers. Although the delivery of chemotherapy appears feasible as part of treatment for sinonasal malignancies, current reported experiences are unable to provide direct comparative evidence of efficacy. The use of chemotherapy remains unproven for other types of histological diagnosis [24].

Lymph node involvement in sinonasal malignancies is less common compared with several other head and neck tumour sites. However, involvement of lymph nodes at presentation is recognised as a poor prognostic factor for regional and distant disease [8, 24, 25], and patients who relapse in regional lymph nodes have an adverse prognosis [8]. In this series only two patient received elective neck irradiation, both for T4N0 squamous cell carcinomas of the maxillary sinus. Four patients developed regional recurrences on follow up; all of these patients had primary cancers of the nasal cavity. The average incidence of neck recurrences in untreated necks following treatment of squamous cell carcinoma of the maxillary sinus is 15 % [6]. The role of elective neck irradiation remains controversial for sinonasal squamous cell carcinomas [3, 6]. The National Comprehensive Cancer Network guidelines recommend elective neck irradiation for T3/T4 squamous cell maxillary carcinomas [26]. A recent meta-analysis has suggested that elective neck irradiation can reduce the rate of nodal recurrence for patients with N0 squamous cell carcinoma of the maxillary sinus [6]. The occurrence of regional nodal recurrence following treatment of nasal cavity cancers suggest that elective nodal radiotherapy could be considered for patients with locally advanced squamous cell carcinoma of the nasal cavity. Factors which need to be considered in evaluating the need for elective neck irradiation for patients on an individual basis include the histological type of malignancy, site, local extent, pattern of potential lymphatic spread and comorbidity.

Distant metastatic failure occurred in 2/43 (5 %) patients; other series including mixed histological diagnoses have reported distant metastases occurring in 18–29 % of patients [2, 4, 8]. These data suggest that although obtaining local control remains a major issue, the potential for distant disease dissemination needs to be considered in designing novel approaches to improve the outcomes of sinonasal malignancies.

One limitation of this series is the absence of robust long term toxicity data. Treatment-related visual impairment may occur with longer term follow up [27]. Toxicity is recorded in an ad-hoc manner without any ophthalmological testing, and as such we are not able to document toxicity in a reliable manner. Importantly, IMRT techniques are now an established method to minimise the risk of optic pathway complications from radiotherapy treatment.

## Conclusions

In summary, definitive or adjuvant radiotherapy provides an effective treatment for sinonasal squamous cell carcinomas. The main pattern of failure remains local, suggesting the need for investigation of intensified local therapy. Whilst remaining uncommon, the cases of regional failure mean that the merits of elective lymph node treatment should be considered on an individual basis.

## Abbreviations

IMRT: Intensity modulated radiotherapy
GTV: Gross tumour volume
CTV: Clinical target volume
PTV: Planning target volume
LRDFS: Locoregional disease free survival
DMFS: Distant metastases free survival
PFS: Progression free survival
CCS: Cause specific survival
OS: Overall survival
HR: Hazard ratio
